# Power and powerlessness in a pandemic

**DOI:** 10.1038/s41390-021-01480-z

**Published:** 2021-04-16

**Authors:** Miriam R. Fine-Goulden

**Affiliations:** Paediatric Intensive Care Unit, Evelina London Children’s Hospital, Guy’s & St. Thomas’ NHS Foundation Trust, London, UK

‘PAED TO THEATRE ONE!’ barks the voice urgently.

I put down my sandwich mid-mouthful and run to the obstetric operating theatres, pausing in the ante-room to stuff my hair into a theatre scrub cap and grab some gloves. I arrive just in time to get a rushed handover from the midwife. The mother is an inmate at the women’s prison. She has taken some drugs—no one is sure exactly what, but she is known to have an opiate use disorder and is on methadone—and was complaining of abdominal pain this morning. When she was brought into hospital for medical review, the baby’s heart recording looked worrying so she was rushed to theatre for an emergency Caesarean section under general anaesthetic. The baby is delivered and handed to me. She is blue and silent. I hold my breath and rub her with a rough hospital towel. She starts slowly to take some shallow breaths, turns a more reassuring shade of pink, and I exhale. Her mother is unconscious. I wrap up the baby as snugly as I can and wheel her down to the neonatal unit to begin the long treatment course for opiate withdrawal, knowing that she will be put up for adoption as her five older siblings have before her.

‘PAED TO ROOM FIVE!’ comes the shrill call, 20 minutes later.

I sprint down the corridor and burst into the dimly lit delivery room in which soft, soothing music is playing. A doula sits on one side of the be-stirruped mother offering calm reassurance in hushed tones, while the baby’s father holds the mother’s other hand and mops her brow. The baby is pulled out and placed onto her mother’s chest. She is embraced by three pairs of loving arms as her mother whispers over and over into her tiny pink ear, ‘I love you, I love you, I love you…’

Two little girls born in the same corridor, in the same hour, who are nevertheless worlds apart. When I consider the stark contrast in their life prospects, I can’t hold back the tears.

Sixteen years on from this training post in Neonatology, I still can’t recount this story without crying. As a fully-fledged Paediatric Intensivist, I have witnessed the entire spectrum of life and death and deal daily with the pain and suffering of children and families. Yet, the feeling of helplessness I experienced on that day—in the first job of my medical career—has never left me.

Before committing to a career in intensive care, I worked as the duty doctor for ‘Child Protection Medicals’ during my paediatric training. My job was to carry out a full medical examination on children brought in by police officers and social workers, to look for and document any physical manifestations of abuse and neglect. The teenage girl whose modest dress covered countless bruises in the familiar angular shape of wire clothes hangers, inflicted by her father. The 2-year-old found in his pushchair (without a coat) by neighbours in the middle of a winter’s night, down the stairwell outside his sex-worker mother’s apartment, lest he make a noise and disturb a client. The 6-year-old girl with adult-sized bite marks up her leg. The gaunt young siblings found alone in an apartment with no food who couldn’t remember when they last ate. What could I do for them as a doctor? Bear witness to their suffering. Attest to their pain. Mostly I just wanted to hug them, to take care of them. And too their parents—who almost certainly had similar experiences of childhood themselves—and tell them that I wish I could have done something to make their lives better, to make it easier to parent, to provide them with stable families, communities, livelihoods.

I am often asked about my job and why I elected to specialise in intensive care. ‘Oh my goodness, how can you bear it…?’ is typically the first response I receive. ‘Looking after all those sick children! Seeing children die…’ Just as for any career or life choice the answers are myriad. I love the variety, the pace, the physiology, the technology, the team dynamics. Whilst it never gets easier dealing in the currency of grief and anguish, there are also moments of tremendous relief and joy. Thanks to advances in our understanding of critical illness and the medications and technology we have to offer, the vast majority of children I care for get better—even in the most desperate of situations—and for the ones who sadly don’t, I know that if my team and I can’t help them, no one can. We are the end of the line. Yes, there is pain and suffering in paediatric intensive care—don’t get me wrong—but I have drugs! I have machines! I really can do something most of the time. But the pain and suffering out there in the world outside the hospital walls, the sheer depth of the inequity—that is entirely beyond me.

A few years ago I went in search of The People in Charge. I spent a year away from my clinical role working full-time in national healthcare policy and administration. I sat in an office, went to meetings, helped make decisions that would affect the lives of hundreds, thousands, millions… I was gazing through the other end of the telescope, considering the health & wellbeing of an entire population and not the flesh, blood, guts and gore of an individual person. I thought I might be able to discover the answers to the inequity I witnessed in my clinical practice, but instead it just raised more questions.

The Covid pandemic has brought this contrast to the fore. The country is clapping, and whilst I am moved to tears by the sentiment, the gratitude, the public spirit, I also somehow feel like a fraud, undeserving of the accolades. I’m just doing my usual job—albeit in very unusual circumstances. But now the axis of the world has shifted and we find ourselves at the centre. We are main characters in this story. We have costumes. We have superpowers.

While I stand at a patient’s bedside I'm shielded from thoughts of the outside world. Our clinical roles are so busy, the hours so fraught, the job so all-consuming that other concerns pale into insignificance. And yet when I leave the hospital they loom large. As I walk through the deserted streets of the local neighbourhood, I wonder what is going on behind those closed doors. With schools shut, I wonder how many children are getting a decent meal. With money tight, tensions high and no means of escape, I wonder how many are getting hurt.

I feel so privileged to do a job that enables me to help save lives, ease pain and suffering, and offer comfort and reassurance, but I have never felt more acutely aware of the limits of my powers. As I am surrounded by images depicting me as a superhero, I have never felt less able to save the world.

